# Delving Deep into Crossmodal Integration

**DOI:** 10.1523/JNEUROSCI.0988-18.2018

**Published:** 2018-07-18

**Authors:** N. Alex Cayco-Gajic, Yann Sweeney

**Affiliations:** ^1^ Department of Neuroscience, Physiology and Pharmacology, University College London, London WC1E 6BT, United Kingdom, and; ^2^ Department of Bioengineering, Imperial College London, London SW7 2AZ, United Kingdom

“Don't you wonder sometimes

'Bout sound and vision?”

—David Bowie

To create a coherent representation of the world, the brain must consolidate information across different sensory modalities. This process, called *multisensory integration*, is key for the meaningful perception of objects and experiences (Maddox et al., 2015). Consider, for instance, how disconcerting it is to watch a film in which the audio and video are slightly out of sync. Traditionally, it was believed that information from multiple senses was integrated in higher cortical areas, after being processed independently in primary sensory areas. This view has recently been challenged by anatomical and functional evidence of crossmodal integration at even the earliest stages of cortical processing (Ghazanfar and Schroeder, 2006).

What is the computational advantage of multisensory enhancement in primary sensory cortex? Recent imaging studies in mouse visual cortex have shown that concurrent auditory stimuli can enhance visual coding by sharpening tuning and modulating firing rates (Ibrahim et al., 2016; Meijer et al., 2017). Moreover, activating auditory and somatosensory cortices elicit similar responses in visual cortex, indicating that the mechanism behind crossmodal integration may be broadly similar across non-primary modalities (Iurilli et al., 2012). There is also considerable evidence of visual and somaesthetic modulation of activity in auditory cortex (for review, see Ghazanfar and Schroeder, 2006). In this case, however, several basic questions remain unanswered, including: what nonauditory features are represented in auditory cortical neurons, how is that information integrated into local cortical circuits, and what effect does this have on auditory processing? To address these questions, and to understand the functional role of crossmodal integration more generally, further interrogation of the circuit mechanism is needed. In a recent article in *The Journal of Neuroscience*, Morrill and Hasenstaub (2018) took a step toward answering these questions by probing the laminar dependence of visual responsiveness in auditory cortex.

Morrill and Hasenstaub (2018) recorded extracellularly from the auditory cortex of awake mice while presenting either auditory (tone) or visual (flash) stimuli. They observed visually-evoked increases of firing rate in 58% of recordings, in both primary and secondary cortical areas, as judged by frequency tuning and auditory response latencies. The use of laminar probes allowed the authors to isolate the effect in different layers, revealing that the significant majority of visual responses occurred in infragranular layers, with minimal responses in L1–L4.

These findings are timely, as they allow direct comparison with several recent experiments that, by contrast, investigate auditory responses in visual cortex. This comparison reveals a functional asymmetry in audiovisual integration in visual and auditory areas. In mouse primary visual cortex, tones and bursts of noise elicit responses in supragranular as well as infragranular layers (but not in L4; Iurilli et al., 2012). In particular, up to 10% of L2/3 neurons respond to tone presentation alone (Meijer et al., 2017). Conversely, Morrill and Hasenstaub (2018) report that <1% of multiunits in L2/3 of auditory cortex were visually responsive.

The strong functional asymmetry between visual and auditory cortex likely stems from a difference in crossmodal input. To understand the source of this difference, it is necessary to identify the main pathways of visual information in auditory cortex. There are three possible pathways: top-down connections from higher-order multisensory areas, connections from thalamus (either visual or multisensory regions), and lateral connections from visual cortex. In rodents, anatomical connections have been observed from all three of these candidates (Ghazanfar and Schroeder, 2006; Banks et al., 2011; Tyll et al., 2011). In the case of lateral connections, there is a striking imbalance between auditory and visual corticocortical projections. For example, a recent tracing study in mice found that, despite significant projections from primary auditory cortex to primary visual cortex, projections in the reverse direction were absent (Ibrahim et al., 2016). Instead, auditory cortex receives input from secondary visual cortex (Banks et al., 2011). Even from these areas, however, an overall asymmetry is apparent. A quick calculation from the Allen Mouse Brain Connectivity Atlas reveals that auditory cortical regions send a greater fraction (by an order of magnitude) of their outgoing projections to visual regions than the converse (Oh et al., 2014). Moreover, the timing of visual responses in auditory cortex is not fast enough to implicate direct projections from early visual cortex as the predominant channel for visual information. For example, Iurilli et al. (2012) reported that activation of auditory cortex elicited responses in visual cortex with a latency of 6 ms. In contrast, Morrill and Hasenstaub (2018) measured visually evoked response latencies of multiunits in both auditory cortex (90 ms) and visual cortex (40 ms). This delay is considerably longer than expected for a monosynaptic connection, suggesting that visual information may be coming primarily from multisensory corticothalamic or higher cortical inputs, at least in mice.

To determine what kind of visual information is integrated in auditory cortex, Morrill and Hasenstaub (2018) repeated their recordings in auditory cortex while presenting drifting gratings of varying orientation. Visually responsive single units were significantly less orientation selective than units in visual cortex, suggesting that these units primarily signaled the timing and presence of a visual stimulus, as opposed to specific visual features. This finding supports the idea that timing is particularly important for crossmodal integration. Indeed, recent studies have demonstrated that temporally congruent auditory and visual stimuli (i.e., having the same temporal frequency) preferentially modulate activity in both ferret auditory cortex (Atilgan et al., 2018) and mouse visual cortex (Meijer et al., 2017) compared with incongruent stimuli. Furthermore, it has recently been demonstrated that projections from auditory cortex to primary visual cortex are dominated by neurons that encode the abrupt onset of sounds (Deneux et al., 2018). However, these recent studies contrast with classic electrophysiological studies, which found evidence of precise frequency and spatial information about auditory stimuli in the visual cortex of cats (Spinelli et al., 1968; Fishman and Michael, 1973). One explanation for this disparity may be the fact that cats have more advanced visual processing compared with rodents. Another possibility is that visual responses in mouse auditory cortex contain information about more complex visual stimuli than the gratings tested by Morrill and Hasenstaub (2018). This may be expected considering that mouse auditory cortex receives direct projections from secondary visual cortex (Banks et al., 2011). However, what visual features these regions represent in mice is unknown (Glickfeld and Olsen, 2017).

Finally, a key result of Morrill and Hasenstaub (2018) is that visual information in auditory cortex was almost exclusively found in infragranular layers, especially in L6. This finding shines a light onto the mysterious role of deep layer neurons. In comparison with their more superficial counterparts, less is known about how L6 neurons contribute to sensory processing. This is due in part to the technical difficulty of accessing deep layers, as well as to the heterogeneous morphologies and unusual response properties of these neurons. Previous work in primary auditory cortex of rats (Sakata and Harris, 2009; Zhou et al., 2010) and cats (Atencio et al., 2009) found that L6 pyramidal cells are less feature selective than cells in superficial layers with complex receptive fields and little stimulus information. These properties have made it difficult to understand the role of L6 neurons for representing auditory stimuli. Although there are likely to be cross-species differences, the findings of Morrill and Hasenstaub (2018) may explain these results by pointing to a more complex role for L6 beyond unimodal auditory processing.

The discovery of a subpopulation of visually-responsive cells in L6 suggests that this layer may serve as a gateway for contextual information from other modalities. Two recent studies in V1 further support this hypothesis. Vélez-Fort et al. (2018) found that L6 pyramidal cells could convey head velocity signals inherited via a direct connection from retrosplenial cortex. Similarly, Leinweber et al. (2017) found that L6 received predictive signals about expected visual flow from motor cortex. Morrill and Hasenstaub (2018) complement these studies by showing that audiovisual integration also takes place in L6 of auditory cortex. Crossmodal integration in L6 could therefore be used to control auditory processing based on nonauditory contextual signals, as L6 of visual cortex has previously been shown to perform gain control on superficial populations without changing their preferred orientation (Olsen et al., 2012). More recently, it has been shown that optogenetic activation of L6 of auditory cortex modulates auditory tuning, and that this could control a tradeoff between sound detection and discrimination performance (Guo et al., 2017). Importantly, this behavioral enhancement was highly dependent on the timing between sensory stimulation and L6 spiking. Combined with the results of Morrill and Hasenstaub (2018), this suggests that visual timing information in L6 may enhance auditory processing. Intriguingly, these studies all target the same Ntsr1-Cre transgenic mouse line, in which Cre-expression is limited to L6 corticothalamic neurons (Sundberg et al., 2018). These findings together suggest the possibility that a population of L6 pyramidal cells perform a crucial role by modulating early sensory processing to generate coherent sensory representations.

The recent burst of work on multisensory enhancement in sensory cortex and on L6 pyramidal cells make this an exciting time for unraveling the circuit mechanism underlying crossmodal integration. Morrill and Hasenstaub (2018) have made a key contribution by revealing the laminar specificity of visual information in auditory cortex. Future studies could help to tease apart the circuit mechanisms of crossmodal integration even further; for example, by dissecting the role of local deep-layer inhibitory circuits. New techniques for large-scale characterization of long-range projections will also clarify how crossmodal information is transmitted between regions (Han et al. 2018). Another open question is whether crossmodal signals can be enhanced by multimodal behavioral tasks. In particular, it would be valuable to investigate whether animals trained to detect specific audiovisual combinations develop tuned visual responses in auditory cortex. Evidence from sensory deprivation experiments hints that such a substrate exists for expressing crossmodal plasticity (Bavelier and Neville, 2002). Ultimately, determining the circuit mechanisms behind crossmodal integration will lead neuroscience further toward understanding naturalistic behavior in dynamic, multisensory environments.
